# Enzymatic Preparation of Carrageenan Oligosaccharides and Evaluation of the Effects on Growth Performance, Serum Biochemical Parameters and Non-Specific Immunity of *Crucian carp*

**DOI:** 10.3390/md23020090

**Published:** 2025-02-19

**Authors:** Limin Ning, Zilong Guo, Benwei Zhu

**Affiliations:** 1 College of Medicine, Nanjing University of Chinese Medicine, Nanjing 210023, China; 2 College of Food Science and Light Industry, Nanjing Tech University, Nanjing 211816, China; guozilong@njtech.edu.cn

**Keywords:** crucian carp, carrageenan oligosaccharides, growth performance, serum biochemical indexes, non-specific immunity

## Abstract

Carrageenan oligosaccharides (COSs) possess versatile activities and have drawn increasing attention in recent years. Due to their unique structures, COSs have been considered to be potential antibacterial agents and immune stimulators. Herein, we aimed to efficiently prepare the COSs by using a novel carrageenase CgkA from *Zobellia uliginosa* with high activity and further investigate the effects of dietary supplementation with COSs on the growth performance, serum biochemical parameters and non-specific immunity in Carassius auratus gibelio. The results indicated that the CgkA could effectively degrade the carrageenan into oligosaccharides with DPs of 2–6 and the oligosaccharides exhibited promoting effects on growth performance, serum biochemical index and non-specific immune parameters. After a 6-month feeding trial, the SR (Survival Ratio) was significantly higher in fish fed 0.1% (Diet 1), 0.2% (Diet 2), 0.5% (Diet 3) and 1% (Diet 4) COSs diets than that in the control group (*p* < 0.05). In addition, the supplementation of COSs decreased the malondialdehyde (MDA) content in the serum and increased the activity of lysozyme (LZM), superoxide dismutase (SOD) and catalase (CAT). In conclusion, COSs as a dietary supplement enhance the growth performance and non-specific immunity of crucian carp and their resistance to diseases.

## 1. Introduction

Crucian carp (*Carassius auratus gibelio*) is one of the most important freshwater species providing high-quality protein for people’s diets [1]. Recently, the rapid development and wide applications of the intensification farming model have caused the spread of bacterial diseases, which have resulted in high economic losses and greatly restricted the development of the green aquacultural farming model [2]. Therefore, various kinds of antibiotics have been used to control traditional diseases in aquaculture. However, the prolonged overuse of antibiotics has resulted in drug resistance and drug residue contamination [3]. Therefore, an effective and eco-friendly natural agent is highly desirable for treating bacterial infections and improving the quality of aquatic animals. Recently, many natural materials (such as alginate [4], chitosan [5], arginine [6], phenylalanine [7], sodium butyrate [8] and phytic acid [9]) have been added to diets to improve the growth performance of crucian carp. However, there are some drawbacks for those agents, such as low solubility, high cost, toxicity and poor bioavailability [10]. Carrageenan is the major component polysaccharide extracted from the extracellular matrix of red seaweeds [11]. Structurally, it shares a general backbone of D-galactose with alternating α-1, 3 and β-1,4 linkages with 15% to 40% content of the ester-sulfate group. It possesses many obvious physiological activities such as antioxidant [12], antitumor [13], anti-inflammatory [14], immunomodulation [15], antivirus [16] and antibacterial activities [11,17]. Carrageenan polysaccharide cannot be assimilated and utilized directly due to its high molecular weight, low solubility and high viscosity. It could be degraded using acidic or enzymatic methods into carrageenan oligosaccharides, which exhibit excellent solubility as well as bioavailability and are a potential resource for manufacturing novel functional products [18].

Herein, the dietary effects of COSs on the growth performance and non-specific immunity of crucian carp were investigated. The growth performance, such as survival percent, the serum index, including serum total protein (TP), serum total cholesterol (CHO), serum high-density lipoprotein cholesterol (HDL-C) and serum total triacylglycerol (TG) were evaluated. In addition, the non-specific immunity factors such as superoxide dismutase (SOD), catalase (CAT) and serum malondialdehyde (MDA), lysozyme (LYM) have also been investigated.

## 2. Results and Discussion

### 2.1. Cloning and Sequence Analysis of CgkA Gene

The open reading frame (ORF) consisted of 1638 bps and encoded a potential carrageenase of 545 amino acid residues with a theoretical molecular mass of 61.94 kDa. According to conserved domain analysis based on the NCBI database, the deduced protein was classified as belonging to family GH 16 [19]. The protein blast of the deduced amino acid sequence of the CgkA shared 85.56% identity with the κ-carrageenase ZgCgkA (AAC27890.1) from Zobellia galactanivorans [20]. The CgkA contained a typical GH16_kappa_carrageenase module at the N-terminal and a Por_Secre_tail module at its C-terminal [21]. The N-terminal module shared a very high identity (over 95%) with the ZgCgkA from Zobellia galactanivorans [22].

### 2.2. Heterologous Expression, Purification of Recombinant CgkA

**Figure 1:**
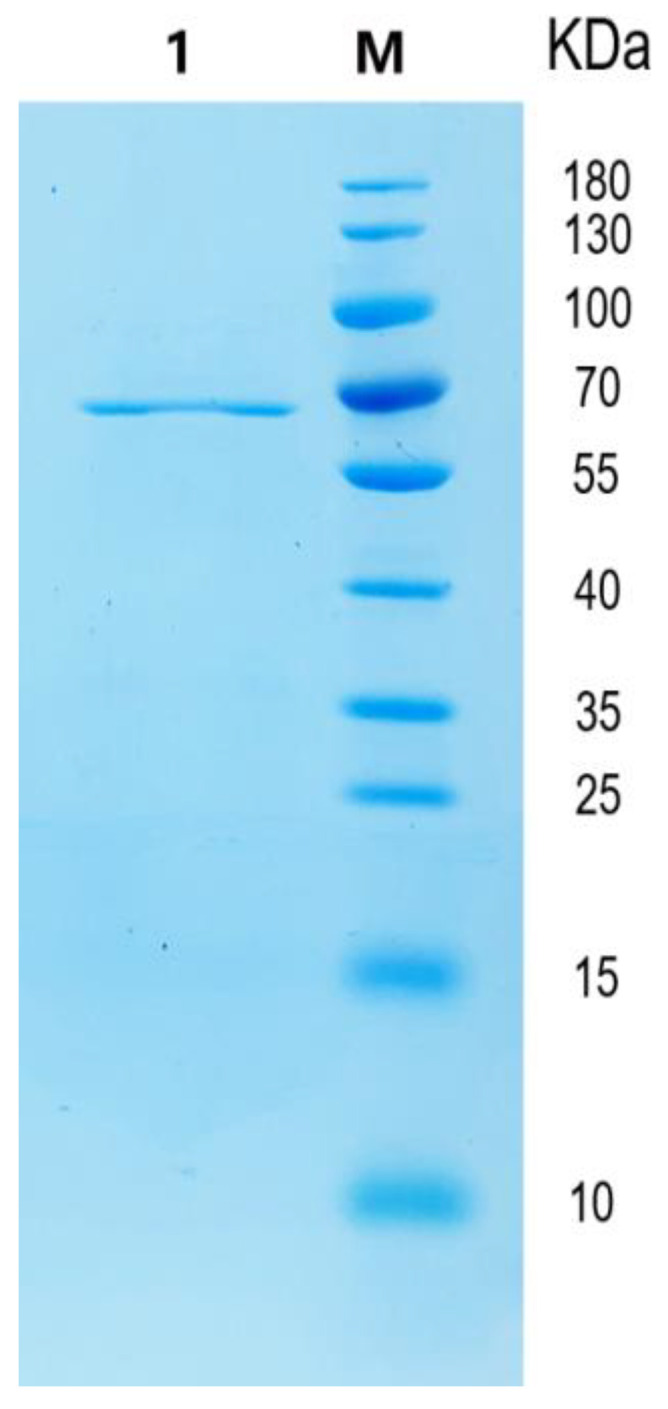
SDS-PAGE analysis of purified CgkA.

The recombinant enzyme was heterologously expressed and further purified using Ni-NTA Sepharose affinity chromatography and analyzed by SDS-PAGE. As shown in Figure 1, a single band with a molecular weight of ~62 kDa was observed in the gel, which is consistent with its theoretical molecular weight. In addition, several other κ-carrageenases have also been recombinantly expressed in *E. coli*, including CgkZ [23], CgkX [24], CgkA [25], Cgk-K142 [26], CgkS [27] and CgkP [28]. These carrageenases form different resources and possess various molecular weights (Mw). Carrageenase CgkNJ [18], CgkAJ5 [28], and CgkX [24] shared a similar smaller Mw of 35 kDa. While Cgk possessed a bigger Mw of 128 kDa [29]. Additionally, the others’ Mws differed from 40 kDa to 70 kDa. Interestingly, the Cytophaga strain produced three isozymes with MWs of 39, 45 and 100 kDa, respectively.

The specific activity of CgkA is 128.76 U/mg, which is higher than other reported carrageenases of the GH 16 family.

### 2.3. Biochemical Characterization of the Recombinant Enzyme

**Figure 2:**
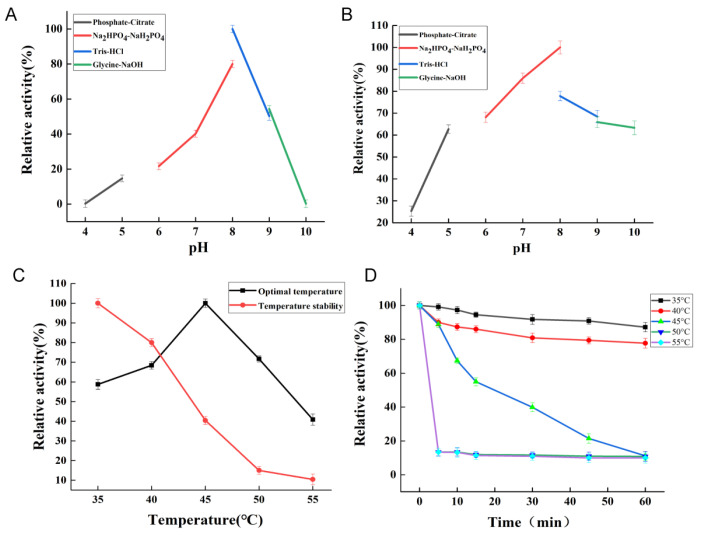
Biochemical characterization of CgkA: (**A**) the optimal pH of CgkA; (**B**) the pH stability of CgkA; (**C**) the optimal temperature and thermal stability of CgkA; (**D**) the thermal degeneration of CgkA.

The biochemical properties have been investigated as shown in Figure 2, the enzyme exhibited maximal activity at 45 °C and pH 8.0. It could retain more than 70% of its activity after being incubated at a pH of 6.0–9.0 below 40 °C. It is similar to most other characterized carrageenases, which also possess an optimal temperature of around 40 °C. For CgkP [28], CgkX [24] and CgkAJ5 [30], they all showed maximal activities at 55 °C and exhibited potential in industrial applications. Meanwhile, for CgkN5-2 [31] and CgkHC4 [32], they showed lower optimal temperatures of 35 °C and 30 °C, respectively. As to the optimal pH, the characterized enzymes all displayed maximal activity at a neutral pH of 6.0–8.0. In addition, the K_m_ and V_max_ of CgkA were determined using Lineweaver–Burk double reciprocal plots. The K_m_ and V_max_ values were calculated to be 0.78 mg/mL and 4.59 U/mL, respectively.

The effects of metal ions on activity are shown in Table S1. The Na^+^ showed activation effects on enzyme activity at the range of 5–150 mM. Specifically, the enzyme activity was boosted by NaCl with different concentrations, where the activity reached the maximum at 150 mM NaCl, being boosted about 2.5 times compared with that without NaCl. Therefore, it was a salt-activated carrageenase and divalent ions such as Mn^2+^, Co^2+^, Zn^2+^, Ni^2+^ and Fe^2+^ showed inactivation effects.

### 2.4. Enzymatic Hydrolysis of Carrageenan and Analysis of Enzymatic Hydrolysate

**Figure 3:**
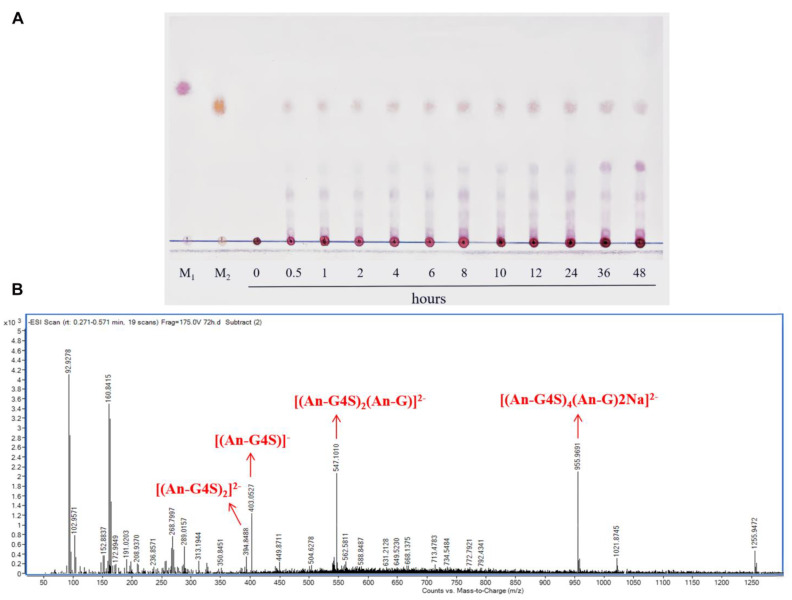
The TLC and ESI-MS analysis of the degradation products of CgkA: (**A**) the TLC analysis of oligosaccharides produced by CgkA, Lane M1, the galactose standard; Lane M2, the carrageenan disaccharide; (**B**) the ESI-MS analysis of oligosaccharides produced by CgkA.

To investigate the degrading ability of CgkA, the course of the hydrolytic procedure was monitored using TLC. As shown in Figure 3A, at the initial stage of hydrolysis (2–12 h), the oligosaccharides with higher DPs (>4) appeared due to the breakdown of the carrageenan endolytically with a large number of oligosaccharides released. Then, the oligosaccharides with higher DPs could be further degraded into the disaccharides and tetrasaccharides. The reported carrageenases all exhibited the endolytic manner to produce even-numbered oligosaccharides [33].

To further identify the component of the hydrolysates mentioned above, ESI-MS was employed. Owing to the presence of sulfate groups on the oligosaccharides, all mass spectral experiments were performed in negative-ion mode. The mass spectra of the products after the reaction for 48 h were shown in Figure 3B. The hydrolysates contained oligosaccharides with different Dps, including disaccharide (403.5 *m*/*z*), tetrasaccharide (394.84 *m*/*z*), and hexasaccharide (547.10 *m*/*z*). Thus, CgkA can degrade κ-carrageenan in an endolytic manner to produce a series of oligosaccharides. CgkA would be a novel potential tool for the production of oligosaccharides with lower Dps.

### 2.5. Homology Modeling and Molecular Docking of CgkA

**Figure 4:**
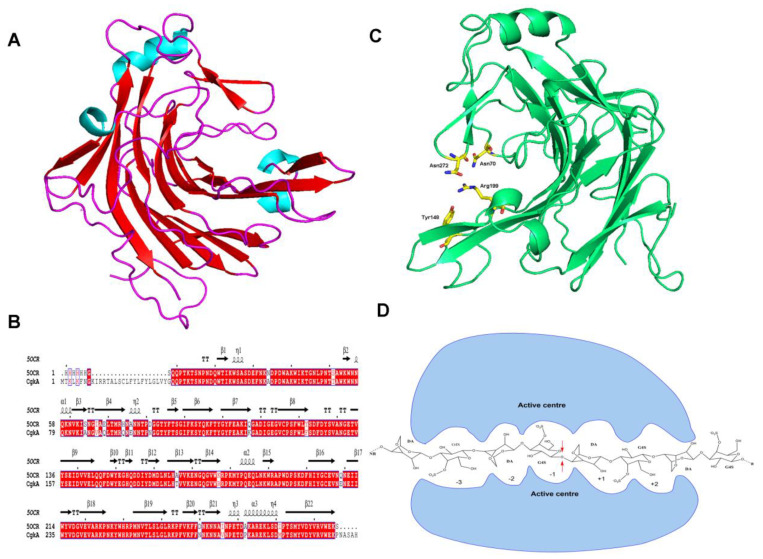
The overall structure, key catalytic residues analysis and action pattern of CgkA: (**A**) the overall structure of CgkA; (**B**) the sequence alignment of CgkA and ZgCgkA_GH16_; (**C**) the catalytic residues in active center of CgkA; (**D**) the action pattern of CgkA.

The three-dimensional modeling results show that CgkA is an almost all-β sheets protein with a globular shape and dimensions. It consists of a β sandwich formed by two main, closely packed and curved antiparallel β sheets of 6 and 7 strands each, creating a deep channel as shown in Figure 4A. It shares a highly similar 3D structure with ZgCgkA (PDB: 5OCR) from Zobellia galactanivorans [23]. The catalytic channel of CgkA is partially closed, forming a tunnel by R199 and N272 (Figure 4B). In addition, the residues for substrate binding and catalytic activity are also highly conserved, as shown in Figure 4B,C. The nucleophile and acid/base catalytic residues of family-16 of glycoside hydrolases have already been unambiguously identified in glucanase and carrageenase [34,35]. The corresponding residues in CgkA are E159 (nucleophile) and E164 (acid/base) (Figure 4B). In addition, D161 could promote the dissociation of the intermediate transition state, and R199 is used to recognize the substrate. Three residues in CgkA (N70, Y148 and N272) were identified as hydrophobic and capable of participating in the binding of substrates [22].

Combining the results of TLC and ESI–MS, octasaccharide was not detected. This indicates that the minimal recognition unit of CgkA is octasaccharide according to the analysis of ZgCgkA [22]. Therefore, the degradation mode of CgkA was predicted (Figure 4D). The CgkA can act on the β-(1,4)-linkage between the subsites +1 and −1 within the carrageenan and produce a series of even-numbered oligosaccharides. At the initial stage of the reaction, CgkA recognizes the smallest unit as octasaccharide and continuously degrades it into disaccharide, tetrasaccharide and hexasaccharide (Figure 4D). Therefore, the action pattern of CgkA is identical to the action mode of ZgCgkA [22].

### 2.6. Growth Performance

The growth performance, feed utilization and survival rate of crucian carp are presented in Table 1. After a 6-month feeding trial, the SR (Survival Ratio) was significantly higher in fish fed 0.1% (Diet 1), 0.2% (Diet 2), 0.5% (Diet 3) and 1% (Diet 4) COS diets than that in the control group (*p* < 0.05). In particular, the SR in Diet 2, Diet 3 and Diet 4 reached 100. However, the WG (Weight gain) and SGR (Specific growth rate) in 0.2%, 0.5% and 1% COS diets decreased compared with the control group. In addition, no significant difference was found in FCR (Feed conversion ratio) and CF (Condition factor) among all experimental groups (*p* > 0.05). The addition of COSs did not improve the growth performance of crucian carp, which may be related to the size of the fish, water temperature, dissolved oxygen and living environment. Liu et al. added different concentrations of fructooligosaccharides into the feed of hybrid tilapia, and the results showed that there was no significant difference in weight gain rate and feed conversion rate among the different groups [36]. Qin et al. reported that dietary chitosan oligosaccharides had no significant effects on mortality, feed conversion rate and weight gain rate of hybrid tilapia [37]. Razeghi et al. found that there was no significant difference in growth performance parameters between the blank control group and the experimental group after mannan oligosaccharides were added to sturgeon feed [38]. Similarly, some experiments have shown that mannan oligosaccharides do not significantly promote the growth performance of other economic fish, including Atlantic salmon [39], Mexican sturgeon [40], hybrid tilapia [41] and golden snapper [42]. Zhang et al. found that dietary chitin oligosaccharides supplementation of 0.6% and 0.8% displayed a significant promotion effect on the weight gain rate of juvenile sea bass, but supplementation at 0.2%, 0.4% and 1.0% exhibited an inhibitory effect on the weight gain rate [43]. The reasons for the above phenomena may be related to the basic diet of fish, animal characteristics, feeding time and feeding environment [43,44].

Oligosaccharides generally affect the growth performance of fish by changing the absorption of intestinal flora, digestive enzymes and minerals [45]. The effect of carrageenan oligosaccharides on the intestinal structure of COSs remains to be further investigated.

### 2.7. Serum Biochemical Parameters Analysis

**Figure 5:**
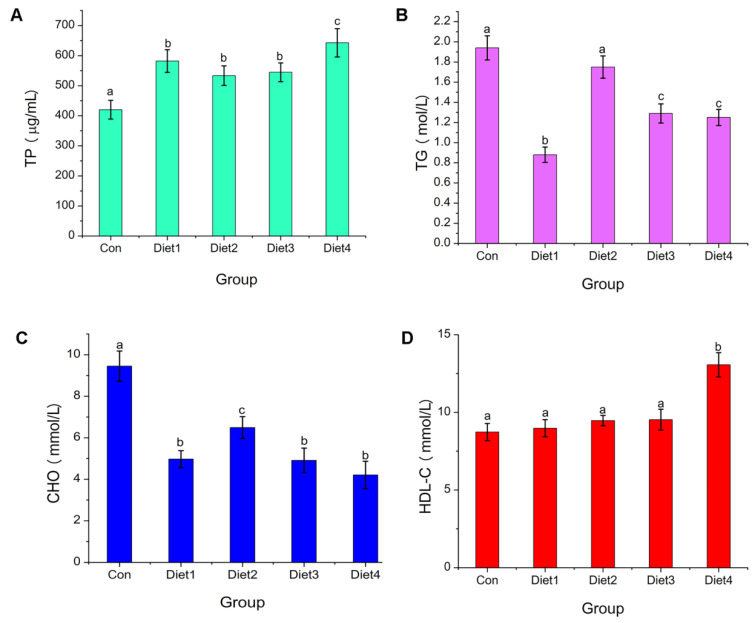
The serum biochemical index in Crucian Carp fed different levels of dietary carrageenan oligosaccharides: (**A**) the TP level; (**B**) the TG level; (**C**) the CHO level; and (**D**) the HDL-C level. Note: TP, Total protein; TG, Total triglyceride; CHO, Total cholesterol; HDL-C, High density lipoprotein cholesterol. Different small letters showed significant difference (*p* < 0.05).

The serum chemical results are summarized in Figure 5. The TP and HDL-C in Diet 4 were the highest among the experiment group, and the two indexes showed a significant dose relationship with the content of dietary oligosaccharides (Figure 5A,D). The CHO and TG in the control group were the highest and exhibited a decreasing trend with the increase in dietary oligosaccharide concentration, but there was no significant difference between Diet 3 and Diet 4 groups (*p* > 0.05). Fish fed with supplemented diets had lower TG and CHO (Figure 5B,C). The level of TP reflected the immune stress state of the fish, and the TP content of fish in the state of disease will decrease [46]. Singh et al. found that adding chitin oligosaccharides to the diet could increase the content of TP in the serum of rainbow trout. Herein, the TP content of the Diet 4 group was significantly (*p* < 0.05) higher than that of other experimental groups, reflecting strong immunological activity [47]. The addition of COSs significantly decreased the content of CHO and TG in the serum and significantly increased the content of HDL-C in the serum. This is similar to the results of Acanthopagrus schlegeli reported by Li [48]. The increase in serum TP and HDL-C contributes to the metabolism of body fat, which could well explain the phenomenon of the gradual decline of the weight gain rate with the increase in dietary oligosaccharide concentration. The reason may be that the sulfuric acid groups in carrageenan oligosaccharides affect the number of receptors of low-density lipoprotein and indirectly play a role in raising the level of HDL-C.

### 2.8. Serum Immune Parameters Analysis

**Figure 6:**
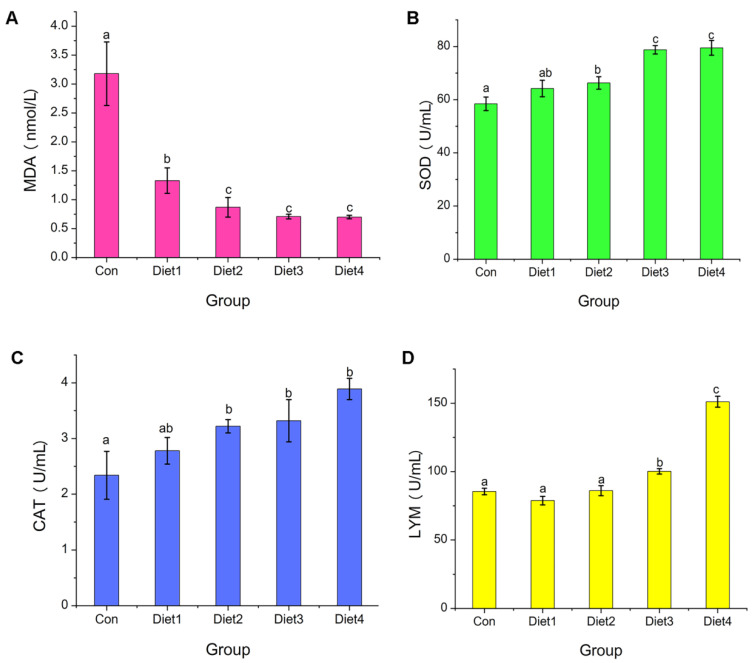
The non-specific immune index in Crucian Carp fed different levels of dietary carrageenan oligosaccharides: (**A**) the MDA level; (**B**) the SOD activity; (**C**) the CAT activity; (**D**) the LYM activity. Note: MDA, Malondialdehyde; SOD, Superoxide dismutase; CAT, Catalase; LYM, Lysozyme. Different small letters showed significant difference (*p* < 0.05).

The result of immune-related parameters in the plasma is shown in Figure 6. Dietary supplementation of COSs decreased the MDA content in the serum (Figure 6A), and increased the activity of LZM, SOD and CAT (Figure 6B–D). The LZM activity was increased with the increasing dietary COSs supplemented, with the highest activity in the Diet 4 group, which is contrary to the MDA content. The highest LZM activity and lowest MDA content were both found in the plasma of fish with Diet 4 diet, which was significantly different from the control group (*p* < 0.05). The crucian carp, as a lower vertebrate, possessed non-specific immunity as the main immune system. The contents of superoxide dismutase (SOD), catalase (CAT), lysozyme (LZM) and malondialdehyde (MDA) in serum are important indicators to measure non-specific immunity of fish [48].

SOD is one of the important antioxidant enzymes in the fish, which can effectively remove the accumulated oxygen free radicals [49]. MDA is one of the main products of membrane lipid oxidation, and its content indirectly reflects the degree of tissue cell damage [50]. LZM is an alkaline enzyme capable of hydrolyzing polysaccharides in bacteria and can induce bacterial dissolution by destroying β-1,4-glucoside bond in bacterial cell walls [51,52]. CAT, as an enzyme scavenger, can effectively remove hydrogen peroxide in the body, thereby protecting cells from invasion [52]. Studies have shown that functional oligosaccharides can act as immune stimulators to regulate their non-specific immune defense system. Li et al. reported that the addition of mannan oligosaccharides to feed could significantly increase the activities of SOD and LZM in the serum of tilapia [53]. Lin et al. found that a supplement of chitin oligosaccharides in the feed could effectively improve the activity of LZM in the serum of carp [54]. Yu et al. discovered that the addition of 0.2–0.3% konjac mannan oligosaccharides to the diet can significantly increase the activities of SOD and LZM in the serum of *Pseudobagrus fulvidraco* [55]. As shown in Figure 6, the activities of serum SOD, LZM and CAT displayed a significant dose relationship with the concentration of COSs, and the activities reached the maximum value when the oligosaccharide supplemental level was 1%, which was significantly (*p* < 0.05) higher than that of the control group.

These results indicate that carrageenan oligosaccharides as a feed additive can significantly improve the non-specific immunity of experimental fish, which is consistent with the increasing survival rate of Carassius auratus during feeding.

## 3. Materials and Methods

### 3.1. Strains and Culture Conditions

The crucian carp (*Carassius auratus gibelio*) was purchased from Nanjing Tongwei Aquatic Technology Co., Ltd. (Nanjing, China). The κ-carrageenan (viscosity: 5–25 mPa.s, solubility: 0.3% in H_2_O) was purchased from Sigma-Aldrich. *Escherichia coli* DH5α and *E. coli* BL21 (DE3) were used for plasmid construction and gene expression, respectively. These strains were cultured in Luria-Bertani (LB) broth or on LB broth agar (LB broth supplemented with 1.5% agar) containing 100 μg/mL ampicillin.

### 3.2. Gene Cloning and Sequence Analysis

In order to clone the gene *CgkA*, the degenerate primers were designed according to the amino acid sequences of the family 16 glycoside hydrolase (Accession number: WP_303400356.1) from *Zobellia uliginosa* [19]. The PCR products were purified and sequenced using Sangong Biotech Co., Ltd. (Shanghai, China). The ORF was analyzed by ORF Finder, and the conserved domain was predicted using the InterProScan 4 running the HMM Pfam application (http://www.ebi.ac.uk/Tools/pfa/iprscan/, accessed on 6 January 2025). The alignment of protein sequences was performed with NTI-Vector. The online software protein homology/analogy recognition engine V (PHYRE) 2.0 (http://www.sbg.bio.ic.ac.uk/phyre2/html/page.cgi?id=index, accessed on 6 January 2025) was applied to establish the three-dimensional structure of CgkA based on the structure of ZgCgkA (PDB: 5OCR) from *Zobellia galactanivorans* with identity of 85.56% [23].

### 3.3. Expression and Purification of CgkA

The primers for sub-cloning the CgkA gene were designed based on the gene sequence containing *Xho*I and *Nde*I sites as follows. The upstream primer sequence was ATGACCAAACTGAAATTTAACGGCAAAA, while the downstream primer was TTCCACCAGAATTTTTTTGCTCACTTCGCC. The CgkA gene was then sub-cloned and ligated into the pET-21a (+) expression vector. The expression and purification of the recombinant enzyme were investigated as previously described [18]. The active fraction was desalted using a HiTrap™ desalting column (Amersham Biosciences, Buckinghamshire, UK) and analyzed by 12% sodium dodecyl sulfate polyacrylamide gel electrophoresis (SDS-PAGE).

### 3.4. Biochemical Characterization of CgkA

The activity was assayed using the 3, 5-dinitrosalicylic acid (DNS) method [56]. One unit of activity was defined as the amount of enzyme needed to release 1 µg of reducing sugars (D-galactose equivalent) per minute [18]. The enzymatic hydrolysis reaction was conducted in 20 mM Tris-HCl buffer (pH 8.0) containing 0.5% (*w*/*v*) κ-carrageenan for 10 min. One unit of enzyme (U) was defined as the amount of protein needed to release 1 μmol reducing sugar (measured as D-galactose) from κ-carrageenan per minute [23]. The optimal pH of the enzyme was evaluated by incubating the purified enzyme in buffers with different pH (4.0–10.0) under standard assay conditions. The pH stability was characterized by determining the residual activity after the enzyme was incubated in buffers with different pH (4.0–10.0) for 24 h. The optimal temperature of the enzyme was determined at different temperatures (35–55 °C) using standard assay conditions at pH 8.0. The temperature stability of the enzyme was evaluated by measuring the residual activity after the enzyme was incubated at 35–55 °C for 30 min. In addition, the thermally induced denaturation was also investigated by incubating the enzyme at 0–60 °C for 0–60 min. The kinetic parameters of the CgkA towards substrate were determined by measuring the enzyme activity with substrates at different concentrations (0.1–8.0 mg· mL^−1^) under 45 °C and pH 8.0. The K_m_ and V_max_ values were calculated by double-reciprocal plots of Lineweaver and Burk [18].

The influences of metal ions on the activity of the enzyme were performed by incubating the purified enzyme at 4 °C for 24 h in the presence of various metal compounds with a concentration of 1 mM. Then, the activity was measured under standard test conditions. The reaction mixture without any metal ions was taken as a control.

### 3.5. Enzymatic Hydrolysis of Carrageenan and Analysis of Products

In order to investigate the processive degrading ability of the enzyme, the hydrolytic system with superfluous substrates was established as follows: 100 mL enzyme (~0.62 mg/mL) was mixed with 10 L 0.5% carrageenan supplemented with 0.01% (*w*/*v*) NaN_3_ and 40 μg/mL of tetracycline, and then the mixture was incubated at 40 °C. The aliquot samples were taken at 0–48 h to determine the amount of reducing sugars by using the TLC analysis as previously described [18]. After incubation, the mixture solutions were boiled for 10 min and then centrifuged at 8000× *g* for 10 min to remove the insoluble materials. The hydrolysates were loaded onto a carbograph column (Alltech, Grace Davison Discovery Sciences, Columbia, MD, USA) to remove salts, and then the eluate was concentrated, dried and redissolved in 1 mL acetonitrile/1 mM NH_4_HCO_3_ (1:1 (*v*/*v*)).

In order to determine the composition and degree of polymerization (DPs) of the products, ESI-MS was employed. In brief, 2 mL supernatant of the redissolved system was loop-injected to Micromass Q-TOF and Q-TOF Ultima instruments (Waters, Manchester, NH, USA) after centrifugation. The oligosaccharides were detected in a negative-ion mode using the following settings. The spray voltage was set at 4 kV, with a sheath gas (nitrogen gas) flow rate of 30 arbitrary units, an auxiliary gas (nitrogen gas) flow rate of 5 arbitrary units, a tube lens voltage of −250 V, a capillary temperature of 350 °C, and a capillary voltage of −48 V. The scan rate was normal, and the type was full, with a microscan number of 3, searching for a mass range of *m*/*z* 200−2000.

### 3.6. Diet Preparation

Carrageenan oligosaccharide prepared in Section 2.5 was supplemented to the diet in four groups, namely, 0.1%, 0.2%, 0.5% and 1% (*w*/*w*). The diet without COS supplementation was used as the control diet. The diet was purchased from Nanjing Tongwei Aquatic Technology Co., Ltd. (Nanjing, China).

### 3.7. Fish Culture and Feeding Trial

The crucian carp were obtained from the fishpond of Nanjing Tongwei Aquatic Technology Co., Ltd. (Nanjing, China). An adjustment of two weeks was provided for the *Carassius auratus gibelio* before the main experiment, according to the previous report [57]. During the adjustment period, fish were fed twice a day (8:00 and 18:00, respectively) with the control diet without COSs. The water was filtered through coral sand, and oxygen was provided by a low-pressure blower. Sufficient oxygen was provided by a lower-pressure blower continually.

Then, fish were selected and completely randomized to 5 fiberglass tanks (300 L, 40 fish per tank, 3 replicates per diet) with an initial body weight of 5.10 ± 0.01 g. During the feeding trial, fish were fed a diet at 5% of body weight twice per day (8:00 and 18:00, respectively), and the feeding amount was slightly changed every two weeks according to the growth of fish and feed intake. The water temperature, pH and dissolved oxygen were measured daily, and total ammonia nitrogen was measured weekly [57]. The temperature and pH of the rearing water were kept at 20 ± 2 °C and pH 7.5 ± 0.5, respectively. The dissolved oxygen was more than 5.0 mg/L. The ammonia nitrogen was lower than 0.01 mg/L, and a natural photoperiod (12/12-h day/night) was used during the trial. The uneaten diet was aspirated by siphoning manually after 40 min of feeding and dried to record the feed intake. One-third of the water in each tank was replaced every three days with tap water. The feeding trial lasted for 6 months.

### 3.8. Sampling

After 6 months of the feeding trial, all fish need to be fasted for 24 h before sampling. The weight and number of fish in each tank were recorded. Ten more fish were randomly taken from each tank and anesthetized with MS-222 (Sigma, St Louis, MO, USA) to measure the body weight and standard length, and blood samples were collected with heparinized syringes, respectively. The blood samples were centrifuged (8000× *g*, 15 min) at 4 °C, and their serum was gathered and stored at −80 °C until analyzed. All experimental procedures were conducted in conformity with institutional guidelines for the care and use of laboratory animals at Nan University of Chinese Medicine, Nanjing, China, and conformed to the National Institutes of Health Guide for Care and Use of Laboratory Animals (publication no. 85–23, revised 1985) [57].

### 3.9. Chemical Analysis

The listed formula calculated the growth performance and morphology parameters of crucian carp [57]:

IBW (g), initial mean body weight;

FBW (g), final mean body weight;

WG (Weight gain) (%) = [final mean weight (g) − initial mean weight (g)] × 100%;

SGR (Specific growth rate) = 100 × (ln final weight − ln initial body weight)/180 days.

FCR (Feed conversion ratio) = final mean feed intake (g)/final mean fish weight gain (g) × 100%;

SR (Survival Ratio) (%) = 100 × final fish number/initial fish number.

CF (Condition factor) = 100× body weight (g)/body length (cm)^3^

### 3.10. Biochemical and Non-Specific Immune Parameters

The serum was thawed and analyzed with an automatic biochemical analyzer (Hitachi 7170; DAICHI, Tokyo, Japan). The items, including total protein (TP), total triglyceride (TG), high-density lipoprotein cholesterol (HDL-C) and cholesterol (CHO) were tested. The determination of superoxide dismutase (SOD), lysozyme (LZM) and malondialdehyde (MDA) was referred to the method described by Zhang et al. [57]. All the parameters were analyzed by following the protocols of the kits (Nanjing Jiancheng Bioengineering Institute, Nanjing, China). In order to ensure the accuracy of the experiments, all experiments were performed with three replicates.

### 3.11. Statistical Analysis

Analysis of variance (ANOVA) and LSD tests were run to compare different treatments using the SPSS statistical package version 22.0 (SPSS Inc., Chicago, IL, USA). The Kruskal–Wallis test was administrated when inhomogeneous variance appeared. Mean values were considered significantly different at *p* < 0.05. All columns in this study were plotted with GraphPad Prism 5.0.

## 4. Conclusions

The results indicated that the CgkA could effectively degrade the carrageenan into oligosaccharides with DPs of 2–6 and the oligosaccharides exhibited promoting effects on growth performance, serum biochemical index and non-specific immune parameters. After a 6-month feeding trial, the SR (Survival Ratio) was significantly higher in fish fed 0.1% (Diet 1), 0.2% (Diet 2), 0.5% (Diet 3) and 1% (Diet 4) COSs diets than that in the control group (*p* < 0.05). In addition, the supplement of COSs decreased the malondialdehyde (MDA) content in the serum and increased the activity of lysozyme (LZM), superoxide dismutase (SOD) and catalase (CAT). In conclusion, COSs as a dietary supplement enhance the growth performance and non-specific immunity of crucian carp and their resistance to diseases. Therefore, the carrageenan oligosaccharides were efficiently prepared by using a novel carrageenase CgkA, which has been systematically characterized and elucidated in biochemical properties and action patterns. The dietary supplement of COSs increased the growth performance and non-specific immunity of crucian carp. The results indicate that COSs can be used as an immunostimulant to improve the growth and immunity of crucian carp, and this finding could provide some useful information and guide in the aquaculture industry. Considering the cost of preparation of COS on a large scale, it is still challenging to realize the applications of COS as an additive in fodder for fresh fish. This is the common drawback of developing novel natural and green additives in aquaculture. In view of this situation, we further aimed to improve the catalytic performance of the enzyme by molecular modification such as rational design or immobilization to efficiently prepare the COS at lower cost.

## Figures and Tables

**Table 1:** Growth-related parameters of crucian carp fed different levels of dietary carrageenan oligosaccharides.

Growth Performance	Control	Diet 1	Diet 2	Diet 3	Diet 4
IBW (g)	5.03 ± 0.02 ^a^	5.21 ± 0.02 ^a^	5.17 ± 0.02 ^a^	5.09 ± 0.01 ^a^	5.11 ± 0.03 ^a^
FBW (g)	32.16 ± 0.36 ^a^	39.33 ± 0.79 ^b^	34.47 ± 0.58 ^a^	31.93 ± 0.47 ^ac^	28.28 ± 0.76 ^c^
WG (%)	539.36 ± 35.33 ^a^	654.89 ± 28.65 ^b^	566.73 ± 31.56 ^a^	527.31 ± 12.45 ^a^	453.47 ± 19.33 ^c^
SR (%)	65	75	100	100	100
SGR (%)	2.60 ± 0.03 ^a^	2.68 ± 0.03 ^a^	2.60 ± 0.02 ^a^	2.57 ± 0.05 ^a^	2.49 ± 0.02 ^a^
CF (%)	2.18 ± 0.02 ^a^	1.99 ± 0.03 ^a^	2.11 ± 0.06 ^a^	1.97 ± 0.08 ^a^	2.03 ± 0.05 ^a^
FCR	1.97 ± 0.06 ^a^	1.98 ± 0.09 ^a^	2.06 ± 0.07 ^a^	1.89 ± 0.03 ^a^	1.92 ± 0.02 ^a^

Note: IBW: initial mean body weight; FBW: final mean body weight; WG: weight gain; SR: survival rate; SGR: specific growth rate; CF: condition factor; FCR: feed conversion ratio. There was no significant difference in the same row of data without superscripts (*p* > 0.05). Different small letters (a, b and c) showed significant difference (*p* < 0.05).

## Data Availability

The data that support the findings of this study are available upon reasonable request.

## Supplementary Materials

The following supporting information can be downloaded at: https://www.mdpi.com/article/10.3390/md23020090/s1, Table S1. Effects of metal ions on enzyme activity.
