# Supplementary material for: Enzymatic Preparation of Carrageenan Oligosaccharides and Evaluation of the Effects on Growth Performance, Serum Biochemical Parameters and Non-Specific Immunity of Crucian carp
Source: Mar Drugs. 2025 Feb 19;23(2):90. doi: 10.3390/md23020090 (PMC11857235; doi:10.3390/md23020090)
Supplement: Supplementary file 1 [file marinedrugs-23-00090-s001.zip › marinedrugs-3484878-supplementary.pdf]

Table S1. Effects of metal ions on enzyme activity.

Metal Ions	Concentration（mM）	Relative Activity（%）
Control	0	100
K^+	1	98.5
Na^+	1	97.7
Na^+	5	130.4
Na^+	50	238.1
Na^+	150	257.5
Co^{2+}	1	59.7
Fe^{3+}	1	71.6
Cu^{2+}	1	44.5
Zn^{2+}	1	29.6
Ni^{2+}	1	78.7
Ca^{2+}	1	99.8
Mn^{2+}	1	62.5
